# National Beef Quality Audit–2016: assessment of cattle hide characteristics, offal condemnations, and carcass traits to determine the quality status of the market cow and bull beef industry

**DOI:** 10.1093/tas/txx002

**Published:** 2018-01-22

**Authors:** McKensie K Harris, L Clay Eastwood, Courtney A Boykin, Ashley N Arnold, Kerri B Gehring, Daniel S Hale, Christopher R Kerth, Davey B Griffin, Jeffrey W Savell, Keith E Belk, Dale R Woerner, Josh D Hasty, Robert J Delmore, Jennifer N Martin, Ty E Lawrence, Trenton J McEvers, Deborah L VanOverbeke, Gretchen G Mafi, Morgan M Pfeiffer, Ty B Schmidt, Robert J Maddock, D Dwain Johnson, Chad C Carr, Jason M Scheffler, T Dean Pringle, Alexander M Stelzleni

**Affiliations:** 1 Department of Animal Science, Texas A&M AgriLife Research, Texas A&M University, College Station, TX; 2 Department of Animal Sciences, Colorado State University, Fort Collins, CO; 3 Beef Carcass Research Center – Department of Agricultural Sciences, West Texas A&M University, Canyon, TX; 4 Department of Animal Science, Oklahoma State University, Stillwater, OK; 5 Department of Animal Science, University of Nebraska-Lincoln, Lincoln, NE; 6 Department of Animal Sciences, North Dakota State University, Fargo, ND; 7 Department of Animal Sciences, University of Florida, Gainesville, FL; 8 Department of Animal & Dairy Science, University of Georgia, Athens, GA

**Keywords:** audit, bull, carcass, cow, hide, offal

## Abstract

To continue the series that began in 1994, the National Beef Quality Audit (**NBQA**) – 2016 was conducted to quantify the quality status of the market cow and bull beef sector, as well as determine improvements made in the beef and dairy industry since 2007. The NBQA-2016 was conducted from March through December of 2016, and assessed hide-on carcasses (*n* = 5,278), chilled carcasses (*n* = 4,285), heads (*n* = 5,720), and offal items (*n* = 4,800) in 18 commercial processing facilities throughout the United States. Beef cattle were predominantly black-hided; 68.0% of beef cows and 67.2% of beef bulls possessed a black hide. Holstein was the predominant type of dairy animal observed. Just over half (56.0%) of the cattle surveyed had no mud contamination on the hide, and when mud was present, 34.1% of cattle only had small amounts. Harvest floor assessments found 44.6% of livers, 23.1% of lungs, 22.3% of hearts, 20.0% of viscera, 8.2% of heads, and 5.9% of tongues were condemned. Liver condemnations were most frequently due to abscess presence. In contrast, contamination was the primary reason for condemnation of all other offal items. Of the cow carcasses surveyed, 17.4% carried a fetus at the time of harvest. As expected, mean carcass weight and loin muscle area values observed for bulls were heavier and larger than cows. The marbling scores represented by cull animal carcasses were most frequently slight and traces amounts. Cow carcasses manifested a greater amount of marbling on average than bull carcasses. The predominant fat color score showed all carcasses surveyed had some level of yellow fat. Only 1.3% of carcasses exhibited signs of arthritic joints. Results of the NBQA-2016 indicate there are areas in which the beef and dairy industries have improved and areas that still need attention to prevent value loss in market cows and bulls.

## INTRODUCTION

Certain characteristics and conditions of cattle may impact harvest practices, as well as the overall value of carcasses and offal. These frequently relate to production practices and can be improved through adjustments in management, thereby increasing the value of carcasses and offal.

One such characteristic is hide contamination via mud and manure. Excessive hide contamination necessitates additional resources during harvest to prevent carcass contamination. In addition, hides excessively laden with mud are generally sold at a lower value due to the potential for latent hide damage to occur during the mud removal process (United States Hide, Skin & Leather Association, 2014). Additionally, heavy manure and fecal contamination on hides pose an increased risk of pathogen transfer to the carcass and increased risk of foodborne illness to the consumer (Elder et al., 2000). Therefore, producers who understand the importance of hide condition at the time of harvest are more likely to employ management practices to minimize hide contamination.

Individual animal identification has been an important component of cattle operations for many centuries (Stamp, 2013). In its early stages, identification primarily consisted of branding the hide to identify ownership. Identification methods and reasons have evolved. Identifying animals with ear tags, metal clips, ankle tags, etc., allows producers to keep accurate records, while facilitating traceability of cows, bulls, and their offspring in all segments of production.

Although not visibly apparent when presented for harvest, conditions that contribute to offal condemnation may be related to producer management (Nagaraja and Chengappa, 1998). The previous National Beef Quality Audit (**NBQA**) determined the most frequently condemned offal items originating from cows and bulls to be livers, viscera, and hearts (Nicholson, 2008). In the NBQA-2007, 45% of livers were condemned, of which 14% were abscessed, 7% were contaminated, 6% had flukes, and 5% showed signs of telangiectasis (National Cattlemen’s Beef Association Beef Quality Assurance Program, 2007). According to USDA’s weekly by-product drop-credit report for September 25, 2017, there was a 30% liver condemnation rate in cows harvested (USDA-AMS, 2017). Because of the high incidence of liver and other offal condemnations and subsequent high economic losses, it is important to evaluate trends in offal condemnations over time to determine if changes in management practices have impacted incidence rates.

Historically, cows and bulls were thought to primarily be a source of lean trimmings (Woerner, 2010). Over time, the industry has realized cow and bull carcasses vary in quality, and certain carcasses may receive premiums for increased quality traits, fat cover, and muscle size (Woerner, 2010). Therefore, understanding trends in cow and bull carcass traits, such as fat cover, marbling score, and muscle score, allows producers to determine if value improvements in meat quality have been attained.

Previous NBQAs conducted in 1994, 1999, and 2007 assessed and quantified the quality status of the market cow and bull beef industry (Smith et al., 1994; Roeber et al., 2001; Nicholson et al., 2013). Audits surveyed characteristics of live animals, carcasses, and offal that could affect value in the market cow and bull sector. To assess changes since the NBQA-2007, the NBQA-2016 was designed to evaluate live cattle, carcasses, and offal throughout 2016. Harris et al. (2017) reported findings related to cattle mobility, live animal defects, hide branding, and carcass bruising. Hide characteristics, offal condemnations, and carcass traits determined in the NBQA-2016 are reported in this publication.

## MATERIALS AND METHODS

### General Overview

This was a collaborative project conducted by Colorado State University, North Dakota State University, Oklahoma State University, Texas A&M University, University of Florida, University of Georgia, University of Nebraska-Lincoln, and West Texas A&M University. To ensure data collection could occur in a single year, the 18 predetermined federally inspected beef processing facilities (Table 1) were divided among the universities. A meeting was held with all collaborators to discuss data collection books and procedures for defining hide characteristics, offal condemnations, and carcass traits being surveyed. Collaborators were instructed to collect observations from one-third of the facility’s production over the course of a single day; when the facility operated two shifts per day, data were collected in both shifts.

**Table 1.** NBQA: company and location of live animal, harvest floor, and cooler assessments

Company	Location
ABF Packing	Stephenville, TX
American Beef Packers	Chino, CA
American Foods Group – Cimpl Meats	Yankton, SD
American Foods Group – Gibbon Packing	Gibbon, NE
American Foods Group – Green Bay Dressed Beef	Green Bay, WI
American Foods Group – Long Prairie Packing	Long Prairie, MN
Cargill Beef Packers	Fresno, CA
Cargill Taylor Beef	Wyalusing, PA
Caviness Packing	Hereford, TX
Central Valley Meat Company	Hanford, CA
FPL Foods LLC	Augusta, GA
H&B Packing	Waco, TX
JBS Green Bay	Green Bay, WI
JBS Omaha	Omaha, NE
JBS Plainwell	Plainwell, MI
JBS Souderton	Souderton, PA
JBS Tolleson	Tolleson, AZ
Lone Star Beef	San Angelo, TX

### Hide Characteristics

Hide-on carcasses (*n* = 5,278) were observed for mud, identification type, and color. If present, location of mud was recorded as being observed on legs, belly, side, top line, and tail region, and its amount was classified as small, moderate, large or extreme levels as defined by Savell (2016). The type of identification (ankle tag, barcode, electronic tag, individual tag, metal clip, lot tag, waddle, and “other”) was also recorded. Additionally, primary color was recorded as the color representing at least 51% of the hide. Hide patterns were classified as Holstein-patterned, baldy, roan, brindle, and spots.

### Offal Assessments

Condemnation by the U.S. Department of Agriculture, Food Safety and Inspection Service (**USDA-FSIS**) of heads (*n* = 5,720), tongues (*n* = 5,720), viscera (*n* = 4,800), livers (*n* = 4,800), kidneys (*n* = 4,800), lungs (*n* = 4,586) and hearts (*n* = 4,586) was recorded, and reasons were documented. The incidence and reason for trimming surveyed heads and tongues was also documented. In addition, surveyed heads were recorded as displaying signs of a broken mouth or were classified as a gummer (an animal that had permanent incisors worn down to the gum line). Pneumonia severity was evaluated with mild being 0% to 15% lung tissue consolidation, moderate being 15% to 50% lung tissue consolidation, and severe being 50% to 100% consolidation of the lung. Surveyed viscera in cow carcasses were assessed for fetal presence. When present, approximate fetalage/size was documented as either “early” (less than 150 days or 35.6 cm or less in length) or “late” (over 150 days or more than 35.6 cm in length).

### Carcass Traits

Hot carcass weight (HCW) and loin muscle area (**LM** area; measured with a dot grid) were recorded for selected carcasses (*n* = 4,285). Lean and skeletal maturity, degree of marbling, preliminary yield grade (**PYG**), and kidney, pelvic, and heart fat (KPH) were evaluated for each selected carcass based on the U.S. Standards for Grades of Carcass Beef (USDA, 2016).

Quality grades for cow carcasses were determined using the relationship between maturity and marbling and were reported as outlined in the U.S. Standards for Grades of Carcass Beef (USDA, 2016). Yield grades for all carcasses were calculated by substituting the values recorded for PYG, HCW, LM area, and KPH into the following equation:

$$\begin{array}{l} \text{2}.\text{5}+(\text{2}.\text{5}\times ((\text{PYG}-\text{2})\times 0.\text{4}))+(0.\text{2}\times \%\text{KPH})-\left(0.\text{32}\times \text{LM area},\text{ square inches}\right)\\ +\;(0.00\text{38}\times \text{HCW},\text{ pounds}). \end{array}$$

If any of the variables necessary for calculating a quality or yield grade were not recorded, a grade was not assigned.

Carcass muscle score was evaluated according to the standards outlined by Nicholson (2008) using a 5-point scale with 1 being the lightest muscled and 5 being the heaviest muscled. Fat color was scored using a 6-point scale with 1 being the whitest and 6 being the most yellow, also defined by Nicholson (2008). If present, the number of arthritic joints on each carcass were documented.

### Statistical Analysis

Data were analyzed using JMP Software (JMP, version 10; SAS Institute Inc., Cary, NC) and Microsoft Excel for Mac. Distributions, frequencies, means, SDs, minimums, and maximums were calculated using the Distribution and Summary functions of JMP. A *z*-test was used to determine differences in frequency of the reason for offal condemnations, if any, between 2007 and 2016 (significance was determined at the 0.05 level). A *z*-test was used because of the large number of degrees of freedom.

## RESULTS AND DISCUSSION

### Hide Characteristics

Mud was not observed on the hides of 56.0% of all cattle surveyed, and those with visible mud were most commonly scored as having small amounts (Table 2). When data are presented by gender and type (Table 2), 57.8% of dairy cows, 54.9% of beef cows, 52.8% of beef bulls, and 48.8% of dairy bulls had no visible mud. Legs and bellies were found to have the highest prevalence of mud in cattle surveyed, while mud was least frequently seen on the side, top line, and tail region (Table 3). Only 42.7% of all cattle surveyed in the NBQA-2007 (Nicholson, 2008) did not have mud on their hide, a lower frequency than we observed. The reduction in mud is indicative of the industry’s initiatives to prevent mud contamination in transport and lairage environments, and remove mud from hides before dressing begins. Mud presence on cattle hides creates potential for cross-contamination of food products when skinning and hide-removal are performed on the harvest floor, thus mud is a potential vehicle for pathogens of foodborne significance (Reid et al., 2002). As such, producers, transporters, and processors all have a role in reducing the prevalence of mud by way of housing animals in dry lots, cleaning trailers, and removing excess mud from the hide before harvesting.

**Table 2.** NBQA: percentage of mud observed in cattle surveyed

Amount^1^	All cattle	Beef cows	Dairy cows	Beef bulls	Dairy bulls
	(*n* = 5,239)	(*n* = 2,094)	(*n* = 2,612)	(*n* = 400)	(*n* = 82)
None	56.0	54.9	57.8	52.8	48.8
Small	34.1	35.0	32.0	39.0	42.7
Moderate	8.1	8.1	8.5	6.8	6.1
Large	1.1	0.8	1.4	0.8	1.2
Extreme	0.7	1.2	0.2	0.8	1.2

^1^Pictorial references for mud scores were used as standards throughout the NBQA-2016 (Savell, 2016).

**Table 3.** NBQA: percentage of cattle with mud on various locations of surveyed cattle^1,2^

Location	All cattle(*n* = 2,304)	Beef cows(*n* = 944)	Dairy cows(*n* = 1,101)	Beef bulls(*n* = 189)	Dairy bulls(*n* = 42)
Legs	82.2	81.9	81.7	83.6	90.5
Belly	54.1	46.1	64.3	36.5	59.5
Side	11.4	10.4	12.9	8.5	9.5
Top line	12.1	9.0	14.7	12.2	14.3
Tail region	7.5	5.8	9.0	8.5	4.8

^1^Sample size is only a representation of cattle with mud present. ^2^Percentages do not add to 100% because multiple responses may have been recorded per animal surveyed.

Over 48% of beef cows, beef bulls, and dairy bulls were tagged with a single form of identification, while more than 67% of dairy cows had multiple forms of identification (Table 4). Ear tags specifying individual animal identification were most commonly observed in all cattle surveyed; however, dairy cows had a much higher frequency of electronic tag identification than other cattle (Table 4). The Holstein Association USA initiated a national tag registration system in 2015 that required registered dairy Holsteins to be tagged once at birth and once again at 6 months of age using official USDA identification with an 840 number (Holstein Association USA, n.d.; USDA-APHIS, 2013). The 840 ear tags can be either a visible identification with numbers or may include a radio frequency identification to be used for electronic scanning. The required tagging procedures likely contribute to why dairy cows were more frequently observed to have two forms of identification and were most tagged with an electronic identification tag. Because the 840 ear tags come in both electronic and non-electronic in addition to being shaped like standard-type ear tags, the determination of the frequency of individual identification may have been overestimated, whereas the determination of the frequency of electronic identification may have been underestimated.

**Table 4.** NBQA: percentage^1^ of identification types in surveyed cattle

Identification	All cattle(*n* = 5,242)	Beef cows(*n* = 2,088)	Dairy cows(*n* = 2,621)	Beef bulls(*n* = 397)	Dairy bulls(*n* = 84)
No ID	8.3	11.9	3.2	20.2	17.9
Single ID	38.6	48.3	29.0	50.1	56.0
Multiple ID	53.0	39.8	67.9	29.7	26.2
Identification type					
Ankle	0.7	0.0	1.4	0.0	0.0
Barcode	1.5	0.9	2.3	0.5	0.0
Electronic	13.2	4.0	22.1	3.0	9.5
Ear tag	69.0	54.9	82.9	54.9	61.9
Metal clip	30.0	38.1	26.7	16.1	8.3
Lot tag	23.0	20.3	27.1	16.1	7.1
Waddles	0.2	0.5	0.0	0.3	0.0
Other	26.9	17.8	34.0	23.4	28.6

^1^Percentages exceed 100% due to animals having multiple forms of identification.

Electronic tag utilization in the dairy industry is practical because cows are handled once, if not twice, daily for milking. Tracking a cow for milk yield by way of scanning an electronic tag while she is in the milking parlor each day allows for sophisticated livestock management and increased producer awareness to the productivity of their cow herd (Eradus and Jansen, 1999). This type of sophisticated technology is less pertinent, yet still useful if efficiently implemented, in beef operations. This is most likely another reason for the higher incidence of electronic tags observed in dairy vs. beef cows and bulls.

As seen in Table 5, 68.0% and 67.2% of beef cows and beef bulls, respectively, had a black-colored hide. There has been a dramatic increase in the number of black-hided beef cows and bulls marketed over the last 9 years; in 2007, Nicholson (2008) reported 44.2% black-hided beef cows and 52.3% black-hided beef bulls. Red-hided beef animals in 2016 were the second most prevalent surveyed; 20.8% and 18.7% of beef bulls and beef cows, respectively. Overall, 80.1% of beef bulls and 74.0% of beef cows were solid colored (Table 6). Baldy-patterned hides were identified on 18.4% and 12.8% of beef cows and beef bulls, respectively. Predominant hide pattern and color for dairy cows (94.2%) and bulls (91.0%) resembled the Holstein breed.

**Table 5.** NBQA: percentage^1^ of each primary hide color observed in cattle surveyed

Hide color	All cattle(*n* = 5,232)	Beef cows(*n* = 2,086)	Dairy cows(*n* = 2,621)	Beef bulls(*n* = 399)	Dairy bulls(*n* = 82)
Patterned animal^2^	51.7	0.1	99.3	0.0	98.8
Black	32.5	68.0	0.3	67.2	1.2
White	1.7	3.0	0.1	4.5	0.0
Yellow	0.9	1.8	0.1	1.0	0.0
Red	9.5	18.7	0.5	20.8	0.0
Brown	3.8	5.0	2.8	3.5	3.7
Gray	1.1	1.7	0.2	2.8	0.0
Tan	1.1	1.7	0.6	0.8	3.7

^1^Percentages exceed 100% due to animals being classified as both patterned and having a primary color. ^2^Includes: Holstein-patterned cattle and cattle with a hide that did not have a primary color covering 51% or more of the hide.

**Table 6.** NBQA: percentage^1^ of each hide pattern observed in cattle surveyed

Pattern	All cattle(*n* = 5,106)	Beef cows(*n* = 2,033)	Dairy cows(*n* = 2,554)	Beef bulls(*n* = 391)	Dairy bulls(*n* = 78)
Solid colored	38.6	74.0	5.1	80.1	9.0
Baldy	8.5	18.4	0.0	12.8	0.0
Roan	0.7	0.9	0.1	0.8	0.0
Brindle	1.3	2.7	0.1	1.8	0.0
Spots	2.7	5.5	0.3	4.6	0.0
Holstein	48.8	nd	94.2	nd	91.0
Other	0.4	0.2	0.2	2.3	0.0

**nd**, not determined. ^1^Percentages exceed 100% due to animals being classified by multiple pattern types.

Studies have shown that a premium price is awarded to black-hided feeder cattle (Bulut and Lawrence, 2007; Schulz et al., 2010). In addition, black-hided beef cows have been shown to receive a premium of $1.69/45.5 kg body weight compared to their red-hided counterparts (Glaze et al., 2016). So, whether a producer is utilizing cows that are black to produce premium calves or he/she is culling black-hided cattle, there is opportunity for increased financial returns. This information should not be the primary reason for making culling decisions, but breeding decisions to increase the percentage of black-hided calves should be considered.

### Offal Condemnations

**Figure 1.**
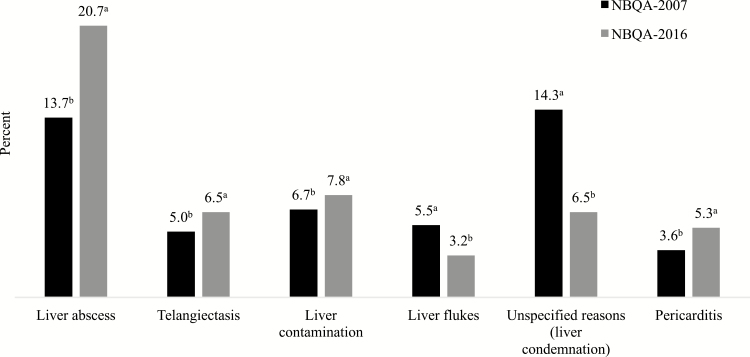
NBQA: frequency distributions for specific offal condemnations from all carcasses sampled in the NBQA-2007 and NBQA-2016. Means within specific condemnation reason with different superscripts differ (*P* < 0.05). Total number of observations for liver condemnations were 4,896 (NBQA-2007) and 4,800 (NBQA-2016). Total number of observations for hearts condemnations were 4,896 (NBQA-2007) and 4,586 (NBQA-2016) (Nicholson, 2008).

Offal condemnations were assessed in all previous NBQAs (Smith et al., 1994; Roeber et al., 2000, 2001; Nicholson, 2008). Frequencies of offal condemnations by USDA-FSIS are reported in Table 7. The liver condemnation rate in the present study was similar to the NBQA-2007, and higher than in the 1994 and 1999 NBQAs. Reasons for liver condemnation included abscesses (20.7%), contamination (7.8%), telangiectasis (6.5%), flukes (3.2%), and unspecified reasons (6.5%) (Fig. 1). There has been an increase (*P <* 0.05) in liver condemnation due to abscesses, telangiectasis, liver contamination, and pericarditis since the NBQA-2007 (Nicholson, 2008), whereas liver condemnations due to flukes and unspecified reasons declined (*P <* 0.05) (Fig. 1). Rezac et al. (2014) observed liver abscesses in 32.2% of the cull dairy and beef cow population they studied. They concluded liver abscesses in dairy cows, in particular, are a result of rapid changes in diet when transitioning from gestation to lactation and the high energy diets that are necessary for maximum milk production (Rezac et al., 2014). A high incidence of liver abscesses may also be seen in cows because of the increased opportunity for the development of “hardware disease” (Rezac et al., 2014). Incidence of liver abscesses may be higher in dairy cows than beef cows, because tylosin phosphate is not labeled for use in lactating dairy cows (Elanco Animal Health, 2017). Producers who elect to feed beef cows prior to harvest to achieve carcass merits eligible for White Fat programs should work with their veterinarian to incorporate tylosin phosphate or other similar products to help mitigate the development of liver abscesses, which in turn will preserve the value of the liver as a by-product.

It is interesting that the frequency of liver abscesses (17.8%) reported in the steer and heifer NBQA-2016 (Eastwood et al., 2017) is lower than that reported in the NBQA-2016 for cows and bulls (20.7%). Being traditionally managed in a feedyard, it would seem steers and heifers have greater exposure to liver abscess development while consuming a high-energy ration. However, the use of antimicrobial feed additives at feedyards under both unregulated situations and regulated with a Veterinary Feed Directive could contribute to the lower rate of abscesses being observed in the steer and heifer population.

Lung condemnations were evaluated for the first time in the present study. Overall, lungs were condemned from nearly one-quarter of the carcasses surveyed (Table 7). Reasons for lung condemnations included: contamination (11.7%), mild pneumonia (4.2%), moderate pneumonia (2.3%), severe pneumonia (1.2%), and other reasons not specified (3.8%) (not in tabular form). There was a 6.2% point increase in the frequency of hearts condemned compared to the previous audit. In the current study, hearts were condemned for contamination (15.5%), pericarditis (5.3%), and other reasons not specified (1.5%). Reasons for viscera condemnations included contamination (10.1%), abscesses (5.1%), ulcers (0.3%), and other unspecified reasons (4.6%) (not in tabular form).

**Table 7.** NBQA: percentages of offal condemnations for carcasses evaluated in NBQA-1994^1^, NBQA-1999^2^, NBQA-2007^3^, and NBQA-2016^4,5,6^

Item	NBQA-1994	NBQA-1999	NBQA-2007	NBQA-2016 (±SEM)
Liver condemnations	30.8	24.1	45.3	44.6 ± 0.007
Lung condemnations	nd	nd	nd	23.1 ± 0.006
Heart condemnations	11.0	7.2	16.1	22.3 ± 0.006
Viscera condemnations	nd	nd	nd	20.0 ± 0.006
Tripe condemnations	44.8	19.2	20.5	nd
Kidney condemnations	nd	nd	nd	10.5 ± 0.004
Head condemnations	11.1	6.7	10.2	8.2 ± 0.004
Tongue condemnations	5.9	9.5	10.0	5.9 ± 0.003

nd, not determined. ^1^NBQA-1994 (Smith et al., 1994). ^2^NBQA-1999 (Roeber et al., 2000, 2001). ^3^NBQA-2007 (Nicholson, 2008). ^4^Total number of observations for liver, viscera, and kidney condemnations were: unknown (NBQA-1994); unknown (NBQA-1999); 4,896 (NBQA-2007); 4,800 (NBQA-2016). ^5^Total number of observations for head and tongue condemnations were: unknown (NBQA-1994); unknown (NBQA-1999); 5,260 (NBQA-2007); 5,720 (NMCBBQA-2016). ^6^Total number of observations for heart and lung condemnations were: unknown (NBQA-1994); unknown (NBQA-1999); 4,896 (NBQA-2007); 4,586 (NBQA-2016).

The rate of head and tongue condemnations was determined during all market cow and bull audits, whereas the rate of heads and tongues that were trimmed before passing inspection was not documented as part of the 1994 and 1999 NBQAs (Smith et al., 1994; Roeber et al., 2000; Nicholson, 2008). In the current survey, heads were condemned for contamination (3.3%), lymph node concerns (1.8%), abscesses (0.9%), and other unspecified reasons (2.2%) (not in tabular form). Heads were trimmed for contamination (0.5%), lymph node concerns (0.3%), abscesses (0.0%), and other reasons (0.2%) (not in tabular form). Tongues were condemned for lymph node concerns (1.4%), contamination (0.6%), hair sore (0.2%), cactus tongue (0.2%), and unspecified reasons (1.8%) (not in tabular form). Tongues were trimmed for hair sore (9.0%), lymph node concerns (4.1%), contamination (2.3%), cactus tongue (1.4%), and other reasons not specified (0.6%) (not in tabular form).

Compared to the NBQA-2007, both head and tongue condemnations have numerically declined (Table 7). Total tongue condemnations have decreased by 4.1% points, while tongues that were trimmed increased by 8.5% points (Nicholson, 2008). There was a decrease in tongues condemned due to cactus tongue (−2.0% points) and hair sore (−1.6% points) compared to 2007, yet an increase in the tongues trimmed due to cactus tongue (+1.4% points) and hair sore (+4.6% points) (Nicholson, 2008). This may indicate a change in tongue inspection protocol by USDA-FSIS; choosing to trim tongues rather than condemn them for hair sore and cactus tongue appeared to be more common for USDA-FSIS inspectors in 2016.

The frequency of broken mouths and gummers observed was 8.5% and 6.2%, respectively (not reported in tabular form). A broken mouth or complete wear of the incisors are very likely causes for culling breeding animals because these animals no longer have the ability to maintain body condition, subsequently reducing their breeding efficacy and functionality.

In the 1999 NBQA, an economic value ($4.49 per animal using 1999 prices) was assigned to the lost opportunity caused by offal condemnations (Roeber et al., 2000). In 2016, this same lost opportunity was computed to provide evidence for improvement in earned value or larger losses in value for offal condemnations. In 2016, offal condemnations cost the industry $2.56 USD per animal. To make accurate comparisons, the prices used to determine the 2016 value loss were used to refigure the value loss in both NBQA-1994 ($1.75 USD) and NBQA-1999 ($1.90 USD) (National Cattlemen’s Beef Association, 2017). This shows that market cow and bull beef producers and/or processors are losing more value due to offal condemnations today than in previous audit years. Therefore, producers should identify ways to minimize and control factors contributing to offal condemnations. Additionally, the scientific community should identify strategies, based on research findings, that allow for producers to maximize profit knowing the frequencies of offal condemnations are at a rate high enough to influence profit earnings.

To determine the incidence of bred cows being harvested, researchers documented the presence of fetuses. Of the cow carcasses surveyed (*n* = 4,692), 17.4% carried a fetus at the time of harvest. This has increased numerically from that reported in the NBQA-2007, where only 10.6% of cows were pregnant at the time of harvest (Nicholson, 2008). During the NBQA-2016, researchers identified 47.1% of the fetuses present (*n* = 815) to be older than 150 d (estimated based on fetal size). By day 38 of pregnancy, the fetus has attached to the uterine wall making pregnancy detection by palpation, ultrasound or blood test very effective (Carpenter and Sprott, 2008). Pregnancy detection by any means is a useful tool in determining when to keep or cull cows based on reproductive performance (Carpenter and Sprott, 2008). A study conducted by the National Animal Health Monitoring System reported that less than 20% of cattlemen check for pregnancy in their cowherd (Bridges et al., 2008). Beef and dairy producers who regularly confirm pregnancies in their herd may cull cows with confirmed pregnancies when the producer believes a cow’s condition is too severe to hold her over until after calving and weaning. However, producers who are not checking for pregnancy before culling are potentially missing an opportunity to capitalize on increased calf crop dollar returns.

### Cooler Assessment

Carcasses surveyed in the NBQA-2016 were evaluated for both quality and yield grade factors outlined by the USDA (Table 8). Beef cow carcasses tended to be slightly fatter, on average, than their counterparts, which may have contributed to beef cow carcasses earning a higher average numerical USDA yield grade than the other carcass types surveyed. Even so, the average adjusted fat thickness determined for beef cow carcasses was not excessively thick. The first NBQA-1994 determined a carcass was “too fat” if assigned a finish score 4 through 9 on a 9-point scale (National Cattlemen’s Beef Association, 1994). Although not a direct comparison, this could be considered the same as identifying any carcass with a PYG over 3.5 (over 1.5 cm of back fat) would be considered “too fat.” Data show that while the average fat thickness for all carcass types is not excessively fat, 17.7% of beef cows, 4.3% of beef bulls, 3.2% of dairy cows, and 1.7% of dairy bulls are “too fat” based on the NBQA-1994 standards (not in tabular form). The criteria outlined in the NBQA-1994 were likely determined because cow and bull carcasses were most often fabricated for use as the lean trimmings source in ground beef mixing (Speer et al., n.d.). Because cow carcasses that meet specific fat type and muscle size specifications are more frequently fabricated into whole muscle primals and subprimals today (Nicholson, 2008; Woerner, 2010), utilization of the NBQA-1994 standards for determining carcasses that are “too fat” may not be appropriate.

**Table 8.** NBQA: mean values for USDA carcass grade traits

Trait	*n*	Mean	SD	Min.	Max.
Beef cows					
USDA yield grade	529	3.1	1.04	0.0	7.4
Adjusted fat thickness, cm	1,718	0.7	0.74	0.0	4.6
HCW, kg	1,728	311.1	86.83	25.5	585.0
LM area, cm^2^	1,132	64.2	17.96	19.35	123.8
KPH, %	628	1.5	1.06	0.0	4.5
Marbling score^1^	1,060	346	131.24	100	970
Lean maturity^2^	1,109	357	131.32	110	600
Skeletal maturity^2^	1,734	497	126.24	100	600
Overall maturity^2^	1,109	443	109.57	150	600
Dairy cows					
USDA yield grade	633	2.8	0.84	0.2	5.8
Adjusted fat thickness, cm	1,708	0.4	0.43	0.0	3.0
HCW, kg	1,714	303.4	73.93	91.8	549.1
LM area, cm^2^	1,133	64.6	15.75	20.0	107.1
KPH, %	696	1.8	1.40	0.0	7.5
Marbling score	1,124	367	142.45	100	950
Lean maturity	1,117	315	127.53	120	600
Skeletal maturity	1,713	413	150.99	110	600
Overall maturity	1,117	387	126.64	145	600
Beef bulls					
USDA yield grade	28	2.4	0.98	0.9	4.8
Adjusted fat thickness, cm	208	0.4	0.49	0.0	3.6
HCW, kg	210	398.4	91.06	115.5	782.3
LM area, cm^2^	141	78.8	16.03	28.38	114.2
KPH, %	33	1.1	0.79	0.0	3.0
Marbling score	129	258	82.99	100	490
Lean maturity	137	380	141.86	160	600
Skeletal maturity	213	422	153.68	140	600
Overall maturity	137	399	136.76	160	600
Dairy bulls					
USDA yield grade	14	2.0	0.7	0.6	2.9
Adjusted fat thickness, cm	58	0.3	0.30	0.0	1.9
HCW, kg	59	373.0	101.68	155.5	665.0
LM area, cm^2^	26	77.5	18.28	36.1	109.7
KPH, %	16	1.2	0.82	0.0	2.5
Marbling score	26	273	89.99	100	440
Lean maturity	26	360	141.27	140	600
Skeletal maturity	59	319	151.75	120	600
Overall maturity	26	360	129.36	160	600

^1^100 = practically devoid^00^, 200 = traces^00^, 300 = slight^00^, 400 = small^00^, 500 = modest^00^, 600 = moderate^00^, 700 = slightly abundant^00^, 800 = moderately abundant^00^, and 900 = abundant^00^ (USDA, 2016). ^2^100 = A^00^, 200 = B^00^, 300 = C^00^, 400 = D^00^, 500 = E^00^ (USDA, 2016).

Bull carcasses had heavier mean carcass weights and larger mean LM area as compared to cow carcasses (Table 8). This is expected as bulls are generally larger than their cow counterparts. Also as expected, due in part to their inherent lack of muscling, dairy cow carcasses were the lightest weight on average. Because of the tendency for dairy cows to be lighter muscled than beef cows, one may expect the average dairy cow carcass LM area to be smaller than the average beef cow carcass LM area. However, data show comparable mean LM areas for the two cow carcass types (Table 8).

Cow carcasses had a greater average amount of marbling than bull carcasses (Table 8). The distribution of marbling scores (Table 9) in both beef cow and dairy bull carcasses indicate that there was an upward shift in marbling compared to 2007. Specifically, the greatest number of carcasses in the NBQA-2016 were assigned a slight marbling score, whereas in the NBQA-2007, the greatest number of carcasses possessed traces marbling (Nicholson, 2008). Dairy cow carcasses and beef bull carcasses had the highest frequency of slight and traces marbling, respectively, which is not different than what was reported by Nicholson (2008). Possibly due to the greater number of cow carcasses surveyed, yet still appropriate to consider, cow carcasses represented all marbling scores—meaning some possessed abundant amounts of marbling, while others had only practically devoid amounts of marbling in the LM. In contrast, bulls only were found to possess practically devoid, traces, slight, and small amounts of marbling.

**Table 9.** NBQA: marbling score frequencies (%) observed in cows and bulls surveyed

Type of animal		Marbling score^1^								
	*n*	PD	TR	SL	SM	MT	MD	SLAB	MAB	AB
Beef cows										
2007^2^	1,057	16.8	27.8	26.2	15.1	6.5	3.3	1.4	0.2	nd
2016	1,129	16.0	22.3	33.6	18.2	5.9	2.2	1.0	0.4	0.3
Dairy cows										
2007	538	8.0	17.2	26.5	22.8	12.2	5.8	3.6	1.7	nd
2016	1,129	11.3	16.3	34.7	23.8	7.3	3.7	2.0	0.4	0.4
Beef bulls										
2007	168	19.2	58.1	15.0	1.2	0.6	0.6	0.0	0.0	nd
2016	138	23.9	50.7	20.3	5.1	0.0	0.0	0.0	0.0	0.0
Dairy bulls										
2007	15	13.3	53.3	20.0	0.0	6.7	6.7	0.0	0.0	nd
2016	33	19.2	30.8	46.2	3.8	0.0	0.0	0.0	0.0	0.0

**nd, not determined**. ^1^USDA (2016): PD = practically devoid, TR = traces, SL = slight, MT = modest, MD = moderate, SLAB = slightly abundant, MAB = moderately abundant, AB = abundant. ^2^National Market Cow and Bull Beef Quality Audit – 2007 (Nicholson, 2008).

Marbling is important because it indicates the palatability of beef and generally has a positive influence on a consumer’s eating experience (Aberle et al., 2012). Because the amount of marbling can increase, lean color can improve, fat can become whiter, and LM areas can become larger as a result of the level of feeding prior to harvest, Woerner (2010) stated, “market cow beef will have an increased influence on beef-eating experiences in the U.S. for years to come.” In a study evaluating the effect of pre-slaughter feeding on carcass characteristics, Allen et al. (2009) reported increased carcass weight, dressing percentage, fat thickness, and marbling scores in dairy cows fed a high concentrate ration for 90 d. Similarly, Schnell et al. (1997) found carcass weight, dressing percentage, and fat thickness to increase when beef and dairy cows were fed for 28 d, while marbling score did not differ between fed and non-fed cows. These studies indicate that exposing market cows to feeding regimes prior to harvest has potential to improve carcass traits that are important for earning producers a premium.

A comparison of USDA carcass traits from the NBQA-2007 (Nicholson, 2008) to the NBQA-2016 is provided in Table 10. Average back fat increased numerically in beef cow and bull carcasses, as well as in dairy bull carcasses. Although back fat in dairy cow carcasses decreased numerically compared to 2007, average LM area increased, which may have contributed to the increased average carcass weight. A numerical increase in average LM area was seen for both dairy bull and beef cow carcasses, whereas average carcass weight increased numerically for beef cows but decreased for dairy bulls. Beef bull carcass weight stayed very consistent in the two audit years, but a drastic numerical decrease (over 12 cm^2^) in average LM area was seen for beef bull carcasses in the NBQA-2016 compared to the NBQA-2007. The beef carcass population possessed a higher average marbling score, whereas the dairy carcass population did the opposite since the NBQA-2007.

**Table 10.** NBQA: means for USDA carcass grade traits from the most recent two NBQA

Trait	NBQA-2007^1^	NBQA-2016^2^
Beef cows		
USDA yield grade	2.6	3.1
Adjusted fat thickness, cm	0.64	0.74
HCW, kg	288.0	311.1
LM area, cm^2^	61.3	64.2
KPH, %	0.3	1.5
Marbling score^3^	314	346
Lean maturity^4^	418	357
Skeletal maturity^4^	525	497
Overall maturity^4^	482	443
Dairy cows		
USDA yield grade	2.8	2.8
Adjusted fat thickness, cm	0.56	0.42
HCW, kg	294.3	303.2
LM area, cm^2^	62.6	64.6
KPH, %	1.1	1.8
Marbling score	388	366
Lean maturity	339	315
Skeletal maturity	489	413
Overall maturity	425	387
Beef bulls		
USDA yield grade	1.6	2.4
Adjusted fat thickness, cm	0.30	0.35
HCW, kg	396.0	396.6
LM area, cm^2^	91.0	78.8
KPH, %	0.2	1.1
Marbling score	228	258
Lean maturity	378	380
Skeletal maturity	414	422
Overall maturity	394	399
Dairy bulls		
USDA yield grade	1.9	2.0
Adjusted fat thickness, cm	0.18	0.26
HCW, kg	420.9	373.0
LM area, cm^2^	75.5	77.5
KPH, %	0.6	1.2
Marbling score	290	273
Lean maturity	354	360
Skeletal maturity	387	319
Overall maturity	367	360

^1^National Market Cow and Bull Beef Quality Audit–2007 (Nicholson, 2008). Total number of observations were: beef cows (*n* = 1,315), dairy cows (*n* = 1,320), beef bulls (*n* = 245), and dairy bulls (*n* = 95). ^2^Total number of observations were: beef cows (*n* = 1,735), dairy cows (*n* = 1,714), beef bulls (*n* = 213), and dairy bulls (*n* = 59). ^3^100 = practically devoid^00^, 200 = traces^00^, 300 = slight^00^, 400 = small^00^, 500 = modest^00^, 600 = moderate^00^, 700 = slightly abundant^00^, 800 = moderately abundant^00^, 900 = abundant^00^ (USDA, 2016). ^4^100 = A^00^, 200 = B^00^, 300 = C^00^, 400 = D^00^, 500 = E^00^ (USDA, 2016).

The mean carcass muscle scores for beef cow (2.4), dairy cow (1.8), beef bull (3.0), and dairy bull carcasses (2.7) indicate carcass muscling varies between beef and dairy breed-type; lower numerical mean muscle scores are reported for dairy cow and bull carcasses than beef cow and bull carcasses (data not in tabular form). Nonetheless, the expectation that dairy cow carcasses yield the least amount of lean muscle is confirmed. The comparison of muscle score frequencies between the NBQA-2007 and the NBQA-2016 is shown in Table 11. Beef cows in both NBQAs were assigned a muscle score 2 most frequently, followed closely by muscle score 3. The highest frequency of dairy cow carcasses reported by Nicholson (2008) were assigned the lowest muscle score, but in the NBQA-2016 carcass muscle appeared to increase, as the highest frequency of dairy cow carcasses were assigned a score 2. In 2016, there was a larger percentage of beef bulls identified closer to the light muscle standard (score 1) than the heavy muscle standard (score 5), something not expected of bull carcasses. Finally, the dairy bull carcass population was assigned a muscle score 2 or 3 more frequently than in 2007, but the overall distribution appears not to have shifted. Because the market cow and bull industry has been, and to an extent still is, driven by production of lean trimmings, it is important for carcasses to be adequately muscled. Producers who are marketing cows and bulls that are inadequately muscled and thin should consider feeding a high-energy ration as it has been shown to improve muscle mass (Schnell et al., 1997). Not only does increased muscle increase the value of cows and bulls, but it may also aid in the reduction of the number of lame animals and non-ambulatory animals; if an animal has enough body weight and muscle mass to be able to walk, it is likely not going to become too weak to walk into the processing facility for harvesting.

**Table 11.** NBQA: muscle score^1^ frequencies (%) compared across the 2007^2^ and 2016^3^ surveys

Muscle score	2007	2016
Beef cows		
1	32.0	21.4
2	31.5	33.5
3	25.3	29.5
4	8.3	11.3
5	2.9	4.3
Dairy cows		
1	53.0	35.3
2	36.8	54.0
3	9.4	9.6
4	0.8	0.9
5	0.0	0.2
Beef bulls		
1	4.9	7.0
2	13.1	24.9
3	30.7	36.2
4	23.4	23.0
5	27.9	8.9
Dairy bulls		
1	11.7	6.9
2	27.7	39.7
3	28.7	34.5
4	19.2	10.3
5	12.8	8.6

^1^1 = light muscled, 5 = heavy muscled. ^2^National Market Cow and Bull Beef Quality Audit–2007 (Nicholson, 2008). Total number of observations were: beef cows (*n* = 1,315), dairy cows (*n* = 1,320), beef bulls (*n* = 245), and dairy bulls (*n* = 95). ^3^Total number of observations were: beef cows (*n* = 1,691), dairy cows (*n* = 1,701), beef bulls (*n* = 213), and dairy bulls (*n* = 58).

The majority of carcasses surveyed in the NBQA-2016 had a fat color score of 2, indicating a slight tint of yellow fat. The mean fat color score for beef cows (3.2) and beef bulls (2.4) was slightly more yellow than dairy cows (2.3) and dairy bulls (2.1). This may be evidence of the difference between beef and dairy cattle management style; beef animals are often raised in range environments consuming primarily roughage-based diets, whereas dairy cattle are managed by feeding greater amounts of concentrate feed. Fat color is an important contributor to the marketability of beef, particularly the middle meats, and thus should be considered to directly affect value of market cows and bulls (Roeber et al., 2000). Fat color scores in excess of 3 have been determined to decrease cull animal value by $2.27 (NBQA-1994), $6.48 (NBQA-1999), and $12.47 (NBQA-2016) USD per carcass (National Cattlemen’s Beef Association, 2017). Some suggest feeding cows a concentrate diet prior to harvest may improve fat color (Woerner, 2010), but others have found no difference in fat color for cows fed concentrate rations prior to harvest (Schnell et al., 1997; Sawyer et al., 2004).

Arthritic joints are another cause of value loss when processing market cows and bulls. Arthritic joints were once determined to contribute to a $9.72 USD per carcass value loss in the NBQA-1999; the loss is now only $1.89 USD per carcass (National Cattlemen’s Beef Association, 2017). Once being a significant concern to the industry, arthritic joints have decreased to frequency of only 1.3%. This is a great improvement from the 11.4% that was observed in the NBQA-1999 (Roeber et al., 2000) and 6.2% in the NBQA-2007 (Nicholson, 2008).

## CONCLUSIONS

Results from the NBQA-2016 indicate the cattle industry has made improvements in hide contamination and carcass traits leading to increased value recovery for producers and processors. In the future, the dairy and beef industries should utilize the findings from the NBQA-2016 to direct research initiatives and formulate producer education efforts for further increasing the quality characteristics of market cows and bulls. Improving producers’ and stakeholders’ knowledge of production practices that can minimize profit loss will allow them to be better equipped to implement management techniques that contribute increased profits and the advancement of the entire beef industry.

*Conflict of interest statement.* None declared.
